# A novel strategy to escape a poor habitat: red-necked grebes transfer flightless young to other ponds

**DOI:** 10.1007/s10211-017-0254-7

**Published:** 2017-02-09

**Authors:** Janusz Kloskowski, Karolina Frączek

**Affiliations:** 1 Institute of Zoology, Poznań University of Life Sciences, Wojska Polskiego 71C, 60-625 Poznań, Poland; 2 Department of Nature Conservation, Institute of Biology, Maria Curie-Skłodowska University, Akademicka 19, 20-033 Lublin, Poland; 3 Urzędowska 252, 23- 200, Kraśnik, Poland

**Keywords:** Brood movement, Habitat selection, Innovative behaviour, Risk taking

## Abstract

Animals confronted with the threat of the death of their offspring may exhibit unusual and risk-prone behaviours. Grebes (Podicipediformes) are water birds which cannot effectively walk, thus unfledged young are assumed to be unable to depart from their natal ponds by land. We provide evidence that red-necked grebes *Podiceps grisegena*, breeding on ponds with scarce food resources, transferred their flightless young (2–4 weeks old) to other, unconnected ponds by land or air. Although a large proportion of breeding grebes in the study area nested on food-poor fish ponds acting as ecological traps, where they suffered significant brood losses, brood movements to new ponds accounted for only 3.3% of such breeding attempts. The infrequency of this strategy may be explained by the lack of suitable territories in close proximity and the high risk of predation or fatal injury. The means of chick transfer remains unclear; the chicks may have followed or been carried by parents shuffling across the pond levees; alternatively, parents may have carried the young on their backs in flight. Our findings indicate that red-necked grebes assess the current level of resources available for chicks and may adopt novel and risky strategies to escape total brood failure.

## Introduction

Novel and risk-prone behaviours in animals may be induced environmentally by the need to cope with unfavourable conditions or environmental change (Reader and Laland 2003). Thus, foraging animals exhibit risk avoidance when resources are abundant but opt for riskier solutions when resources are low (Stephen and Krebs 1986). When their assessment of future habitat conditions has been wrong or resources vary unpredictably during the breeding season, animals may face trade-offs between staying in the original habitat, with a risk of producing low-quality offspring or even losing the young due to limited resources, and exploitation of other habitat patches, with risks associated with crossing hostile habitat. Choosing the latter alternative, parents may either obtain food for the young from distant habitat patches or move their broods to new sites; both strategies may be energetically costly and associated with dangers, such as increased exposure to predators (Monaghan et al. 1994; Leonard et al. 1996; Low et al. 2010).

The red-necked grebe *Podiceps grisegena* is a medium-sized aquatic bird, which, especially in the case of the western Palearctic subspecies, often nests on small, shallow waterbodies, where the shores provide natural habitat and territory boundaries. The species is classified as semi-precocial; upon egg hatching, the family leaves the nest, but the young, vulnerable to chilling, are ‘back-brooded’ for about 2 weeks and remain dependent on parental feeding for 6–8 weeks (Vlug 2002). In Podicipediformes, the ability to walk has been restricted by selection pressure for swimming and diving (Johansson and Lindhe Norberg 2001). Thus, adults are normally not found on land, and when water pathways are not available, the young are not able to depart from the natal ponds on their own until they fledge at 8–9 weeks of age (Vlug 2002). Here we report the risky and unusual behaviour in grebes of transferring flightless young between unconnected water bodies. We hypothesised that grebes which resorted to this novel behaviour faced unfavourable breeding conditions and that the brood movement was a strategy to escape from a poor-quality habitat. A clear dichotomy in the habitat quality (in terms of prey availability) of ponds used by grebes for breeding in the study area provided the opportunity to verify this hypothesis. We predicted that broods would only be moved from poor-quality habitat patches and that chicks would be transferred either to good-quality habitat patches or, in the absence of a good-quality habitat in the proximity of the natal territory, to other poor-quality patches, provided that they are within reach and not yet exploited by conspecifics. Additionally, parent birds should prospect and obtain food from alternative patches prior to undertaking the brood transfer. We also discuss other factors which might necessitate such bold and risky decisions.

## Methods and study area

Fieldwork was carried out between 1994 and 2011 at non-intensively farmed fish ponds dispersed in clusters around the city of Lublin, eastern Poland. In the study area, red-necked grebes nested nearly exclusively on water bodies of two types: ponds stocked with small, young-of-the-year fish (fry) and ponds containing larger, 1-year-old fish (common carp *Cyprinus carpio*). The two pond types differed considerably in habitat suitability. Fry ponds (hereafter considered “good-quality habitat patches”) provided good breeding conditions, as the small fish could be exploited by grebes. In ponds with larger carp (“poor-quality habitat patches”), the fish were unavailable for chicks due to size constraints; grebes feed their young bill-to-bill and do not tear the prey into smaller portions. Moreover, larger-size fish suppressed populations of pond macroinvertebrates and amphibians, which are grebes’ alternative food base. However, since fish were an attractive prey to spring-arriving adults, ponds with larger carp acted as an ecological trap, with very low fledging success of breeding grebes and a large proportion of broods ending in complete loss (Kloskowski 2012). Habitat patches of different qualities were irregularly interspersed; some good- and poor-quality ponds were separated only by levees 6–12 m wide, but occasionally the distance could exceed 0.5 km.

Each year data were collected from 52 to 64 ponds supporting 12–40 red-necked grebe nesting pairs annually. On larger ponds (> 2 ha), more than one pair could breed, defending territories of about 1 ha. Each pond was visited at least weekly to locate breeding territories and nests between mid-April and the end of July. Clutch size, hatching date and fledging success were determined for each nest. From 1996 until 2011, adult and >2-week-old young grebes were captured and metal- and colour-ringed. Body mass was measured to the nearest 1 g with a spring balance and wing length to the nearest 1 mm with a wing ruler to obtain a proxy for wing loading, which was used to indicate the chicks’ ability to fly at the time of brood transfer. Additionally, to assess food availability in the originally selected habitat patches and to determine whether brood transfer augmented the amount of food that the parents could provide to the chicks, 1–8 h observations of brood provisioning were collected from a significant proportion of breeding pairs at 1–5 day intervals for at least the first 4 weeks post-hatching. Prey delivered to chicks was identified to the lowest possible taxonomic level using a ×60 spotting scope or a camcorder with an additional ×12 lens; prey size estimates relative to the grebe’s bill length were converted to approximate wet weight. This enabled later estimation of feeding rates in terms of biomass per hour (more details in Kloskowski 2011) As a measure of food availability in the originally selected habitat patches, we used feeding rates during the first two post-hatching weeks; this period is the most critical for survival of the young (Kloskowski 2003). Repeated measures ANOVA was applied to compare weekly pooled (weeks were entered as a within-subject effect) feeding rates among pairs breeding in good-quality habitat patches, pairs in poor-quality habitat patches which did not switch their territories (only pairs that fledged at least one chick were considered) and pairs in poor-quality habitat patches which moved their broods to other ponds, *n* = 19, 14 and 4, respectively). This analysis was followed by post hoc LSD tests with the level of significance set at 5%. With the exception of one unmarked pair which transferred their chick, the analysis included only pairs with at least one ringed adult.

## Results and discussion

We documented four events (3.3%) out of 120 successful nesting attempts (when at least one chick hatched) on low-quality ponds consistent with transfer of chicks by adult grebes. As predicted by our hypothesis that brood transfer was related to habitat quality, no brood movements from good-quality ponds (*n* = 169 nesting attempts) were recorded. In 1994, in the Samoklęski pond-complex (51° 26′ N–22° 26′ E), an unringed red-necked grebe pair with a chick hatched around 7 June (pair 1) was assumed to have changed their territory based on regular behavioural observations of this pair. This red-necked grebe family was found to disappear from a low-quality nesting pond and appear on an adjacent (i.e. separated only by a levee) good-quality pond on 3 July. Besides food scarcity (see below), the situation of the red-necked grebe pair on the natal pond was worsened by aggressive domination of two neighbouring great crested grebe *Podiceps cristatus* pairs; after the territory switch, the pair shared the new pond with one great crested grebe pair. In 2007, at a fish farm in Kraśnik (50°57′–22°11′), a pair with a single chick hatched on 15 May (pair 2) was observed on a pond adjacent to the nesting pond on 8 June. However, another pair, caring for two chicks hatched around 30 June (pair 3), transferred a ringed chick from the nesting pond to a pond at a distance of about 100 m between 27 and 30 July. The grebes’ route probably included another pond between these two. The other chick remained on the natal pond, which was visited by the parents. In both pairs at least one member was ringed. In 2008, the ringed male from pair 3, now paired to another female in another territory (pair 4), moved with a chick (hatched around 31 May) from the nesting pond (2 ha area) to an adjacent, much smaller (<1 ha) pond on 17 June. On 28 June, the family was again observed in the nesting territory. All Kraśnik pairs nested on low-quality ponds containing large fish and transferred their young to same-type ponds, presumably because more favourable habitats were not available in close proximity (the closest fry pond was >200 m away). No great crested grebe or other red-necked grebe pairs were present on their natal or new brood-rearing ponds. Except pair 4, after brood movement, all pairs stayed on the new brood-rearing ponds until the young attained independence.

Parental feeding rates were significantly lower on low-quality ponds than on good-quality ponds (repeated measures ANOVA, *F*_2,34_ = 11.1, *p* < 0.001) during the early post-hatching period as predicted, but they did not significantly differ between sedentary pairs on low-quality ponds and territory-switching pairs as determined by LSD tests. Provisioning rates per chick significantly increased between the first and second week after brood hatching (*F*_1,34_ = 20.0, *p* < 0.001; good-quality ponds, 3.7 ± 0.5 SE g h^−1^ in the first week vs 6.7 ± 1.0 g h^−1^ in the second week; sedentary pairs, 1.2 ± 0.4 vs 2.4 ± 0.7 g h^−1^; territory-switching pairs, 0.3 ± 0.2 vs 1.2 ± 0.6 g h^−1^). With the exception of pair 4, which obtained most food from other ponds (see below), in the territory-switching pairs, provisioning rates increased (by 0.2–2.2 g h^−1^) between the last week before and the first week after chick transfer. However, since chicks were transferred at different ages (18–28/29 days), it was not possible to determine whether the increase differed from the overall pattern of feeding rate increase with chick age.

In all pairs which changed territories, brood reduction occurred prior to departure from the nesting pond, while all chicks transferred between ponds survived to fledge. The other common characteristic was that prior to the chick transfer, adult grebes used to fly for food to the pond where they later moved with the young. This, again, supports our hypothesis that chick transfer is a strategy to escape a habitat poor in food. The pair 4 adults, which returned to the nesting pond after the territory switch, made foraging flights to other ponds throughout brood rearing, irrespective of which pond they were currently on. Foraging flights to nearby ponds were also recorded in some sedentary pairs breeding in poor-quality habitat patches (in 6 of the 14 pairs) but never in good-quality habitats.

Given their age and poor feeding rates in the natal ponds, the chicks were unlikely to have developed any flight ability at the time of transfer. In pair 3, both young were captured about 2–3 days before one of them was observed with its parents on another pond; at the time of capture (at the age of 25–26 days), they had poorly developed flight feathers and wing lengths of 52 and 64 mm at weights of 345 and 335 g, respectively, which is about 1/3 of the wing length of fledged grebes (the chicks’ parents, 170 and 177 mm at weights of 565 and 695 g, respectively). Hence, the chicks could not fly away from the natal pond by themselves.

The strategy of brood moving was apparently infrequently employed, although feeding levels of pairs which settled and stayed in the poor-quality habitat patches were not significantly higher than in the case of pairs which changed ponds. The frequency may be underestimated due to an unknown number of failed brood movements. However, unrecognized successful transfers are unlikely because breeding territories were located prior to or during the nest stage, or exceptionally in the early post-hatching period, i.e., we had no ‘new’ pairs recorded for the first time when their chicks were over 1 week old. Because adult grebes abandoned the territory after brood loss, territories from which grebe families disappeared prior to the anticipated fledging date were often searched for chick carcasses, which were usually found. Necropsies confirmed that the main agent of mortality was undernourishment (Kloskowski 2003). Obviously, most pairs suffering chick mortality did not transfer their young to other water bodies. Some parents breeding in poor-quality habitat patches obtained additional food for chicks and themselves by means of foraging flights to other ponds (while not attempting to transfer the young), as did the brood-moving pairs prior to the territory switch. Overall, foraging flights did not prevent significant chick mortality in food-poor territories (Kloskowski 2011) and at the same time imposed serious energetic costs on the provisioning adults (Norberg 1981; Ohanjanian 1989). Transferred chicks might survive better not only in good-quality habitat patches but even in hitherto unoccupied (and therefore unexploited) poor-quality habitat patches. Red-necked grebes are food generalists, and some of their prey resources, other than stocked fish, could vary between poor-quality habitat patches. With the exception of the pair which returned to the natal pond after the territory switch, parental feeding rates rose after chick transfer; however, it is unclear whether this was an effective increase due to greater food availability in the new habitat patch or whether feeding rates naturally increased due to chick food demands associated with growth. The explanation for the infrequency of brood movement could be either that the parents of unsuccessful broods were unable to transfer their offspring to better habitats (no such habitats were available in close proximity to the nesting pond; cf. Pöysä and Paasivaara 2006) or that the threat of brood failure was not a strong enough impulse to overcome risk aversion. Also, since a distinct age/size structure is not common in natural fish communities, prey scarcity induced by fish stocking created a new challenge for grebes, and their ability to confront it may be dependent on specific personality traits such as readiness for risk taking (Van Oers et al. 2004).

We assume that the main reason for chick transfer was food scarcity because broods were only moved from poor-quality habitat patches; however, factors other than prey availability are likely to affect habitat suitability as well and thus influence birds’ decisions on territory change. An additional and perhaps decisive catalyst for territory switching could be the aggressive pressure of neighbouring great crested grebes in pair 1 and ringing-related disturbance in pair 3, as pair 3 transferred one chick to another pond shortly after all family members had been ringed during one capture occasion. However, the other brood-moving pairs apparently did not experience interspecific or human disturbance. Red-necked and great crested grebes frequently co-occurred on the same ponds and were interspecifically territorial towards each other, but red-necked grebes were not observed to abandon their nesting ponds at the brood stage.

In precocial birds, brood movements to productive foraging sites and more or less competitive sites can be common, as many precocial species use multiple habitats throughout brood rearing. Also, when their original settling decisions have been poor or the conditions have adversely changed between settlement and the brood stage, precocial parents may correct their original habitat choice and move their young away to gain access to better resources (Kosztolányi et al. 2007). Brood movements, especially across a hostile habitat matrix, jeopardize the brood even in species in which they are a frequent strategy to optimize reproductive success (Blomqvist and Johansson 1995; Leonard et al. 1996; but see Pöysä and Paasivaara 2006). Grebes belong to water bird taxa in which the young are actually non-mobile out of water, and thus during the brood stage, parents are assumed to have no choice but to accept local food conditions, with the exception of territory shifts within larger water bodies. Our results provide the first documented evidence, based on marked individuals, of the transfer of flightless young between unconnected water bodies by water birds not adapted for effective walking. It remains unexplained how the chicks were transferred to the other ponds. The original territorial ponds and the later brood-rearing ponds were not connected by any accessible waterways (e.g. pipes or ditches), so brood movements by water were not feasible. On the ground, red-necked grebes can lunge ahead on the breast and belly by pushing with their feet, or even make a few steps in an erect posture, helping themselves with wing flaps (White 1931; see also Nero et al. 1958). To reach the adjacent pond, the studied breeding pairs only needed to negotiate the levees between ponds, but these were fairly steep (50–70°) and it would be very challenging for the chicks to mount them on their own. One possibility is that the parents covered the overland route with the chicks on their backs. During the early back-brooding period, parents can even dive with their chicks (Vlug 2002). Thus, they may somehow be predisposed to transport chicks on their backs out of water as well. However, grebes are highly vulnerable to both ground and air predators on land. An alternative scenario is that one of the parents carried the young in flight, as proposed in a similar case for the great crested grebe by Coles (1984). Hanzák (1952) reported an anecdotal account of a great crested grebe shot down in flight with a living chick on its back. Grebes need a long running start to take off, and given their high wing loading (Fjeldså 2004), it would pose an extreme physical challenge to become airborne with a 4-week old chick. To avoid dropping the chicks, back-brooding parents press their wings down, which would be impossible during flight. Therefore, both potential routes of brood movement would involve atypical and risky behaviours.

Ecological traps and perceptual errors during habitat selection may require breeding adults to re-evaluate their reproductive options within the breeding season. Notably, the chick transfers occurred only when they were over 2 weeks old, after the broods had already suffered partial losses. Adult grebes were obviously able to assess the current level of food resources (and thus the threat of chick starvation), although not necessarily at the early brood stage. Also, if they are to follow their parents to new territories, chicks may need to attain a certain level of physical development. The chick-transferring events suggest that grebes’ behaviour has some features of anticipation of future needs (Raby et al. 2007), although with clear reinforcement by the current state of brood condition. Ecological traps in novel, man-made habitats create ecological challenges which animals have rarely experienced before and may lead them to adopt novel, innovative behaviours (Sol et al. 2005). Our observations reveal behavioural flexibility in grebes; the change of brood-rearing pond as a parental response to the threat of brood starvation was far from stereotyped behaviour patterns. In whatever manner the chicks were transferred, grebes are poorly adapted to brood movements by routes other than water.
